# From Agro-Food By-Products to Precision Nutraceuticals: Bioactive Compounds, Molecular Mechanisms, Bioavailability and Clinical Translation

**DOI:** 10.3390/foods15203660

**Published:** 2026-10-15

**Authors:** Stefano Ruga

**Affiliations:** Department of Health Sciences, Magna Graecia University of Catanzaro, 88100 Catanzaro, Italy; rugast1@gmail.com

**Keywords:** circular bioeconomy, bioactive compounds, polyphenols, bioavailability, clinical translation, precision nutrition, regulatory science

## Abstract

The valorization of agro-food by-products represents a potential strategy to address both environmental sustainability and the growing demand for innovative health-promoting products. Traditionally considered waste materials, agro-food residues are now recognized as valuable sources of bioactive compounds, including polyphenols, flavonoids, carotenoids, terpenoids, peptides, and other phytochemicals with potential nutraceutical applications. This review aims to provide a critical synthesis of the transition from agro-food by-product recovery to the development of evidence-based precision nutraceuticals. Particular attention is given to the chemical diversity of bioactive compounds obtained from representative agro-food sources, including citrus, olive, grape, tomato, and pomegranate residues, highlighting their biological activities and therapeutic potential. The molecular mechanisms underlying their health-promoting effects are discussed, focusing on key pathways involved in oxidative stress (Nrf2), inflammation (NF-κB), metabolic regulation (AMPK/SIRT1), mitochondrial homeostasis, autophagy/mitophagy, and apoptosis. Beyond the description of biological activities, this review critically addresses the main challenges limiting the translation of agro-food-derived bioactives into effective nutraceutical products, including bioavailability, bioaccessibility, intestinal metabolism, standardization of extracts, variability of raw materials, dose optimization, and the gap between preclinical findings and clinical evidence. The translational bottleneck is examined in detail, emphasizing the need for robust clinical validation and regulatory compliance. Finally, future perspectives are discussed, highlighting the potential of emerging technologies, including precision extraction, omics approaches, artificial intelligence, and personalized nutrition, to overcome current limitations and to enable the development of sustainable, safe, and effective nutraceutical interventions. By integrating advances in food science, molecular pharmacology, and human health research, this review provides a comprehensive perspective on how agro-food by-products can be transformed into scientifically validated nutraceutical platforms, supporting the development of sustainable and personalized strategies for disease prevention and health promotion. Despite promising preclinical evidence, clinical validation remains limited: most human studies are small, of short duration, and none have evaluated hard clinical endpoints.

## 1. Introduction

### 1.1. Circular Bioeconomy

The contemporary food system is confronting a critical paradox: while a substantial portion of the global population suffers from malnutrition, a staggering one-third of all food produced for human consumption is lost or wasted annually along the supply chain [1]. This inefficiency not only represents a profound ethical failure but also exerts immense pressure on environmental sustainability, contributing significantly to greenhouse gas emissions, land degradation, and biodiversity loss. The prevailing linear economic model, based on a “take-make-dispose” pattern, has proven to be both environmentally and economically unsustainable. In response, the concept of a circular bioeconomy has emerged as a transformative paradigm, advocating for a systemic shift towards resource efficiency, waste minimization, and the creation of closed-loop systems where by-products and residues are re-introduced into the production cycle as valuable secondary raw materials [2]. This approach is gaining traction not only as an environmental necessity but also as a strategic economic opportunity.

While recent reviews have addressed individual aspects of this field, such as the recovery of bioactive compounds [3], the circular valorization of by-products [4], or the health effects of specific residues [5], a comprehensive synthesis that critically examines the continuum from by-product recovery to clinically validated precision nutraceuticals is still lacking. This review addresses this gap by integrating chemical, mechanistic, bioavailability, and translational perspectives into a single analytical framework. The primary question guiding this work is: what are the principal scientific and regulatory barriers that currently prevent agro-food-derived bioactives from achieving clinical translation, and how can emerging technologies address them? To ensure transparency in evidence selection, a narrative review approach was adopted. Literature was identified through systematic searches of PubMed, Scopus, and Web of Science, covering the period from January 2000 to July 2026. Search terms included combinations of ‘agro-food by-products’, ‘bioactive compounds’, ‘polyphenols’, ‘bioavailability’, ‘clinical translation’, ‘precision nutrition’, and ‘regulatory’. Priority was given to original research articles and systematic reviews, with clinical trials and human intervention studies considered where available. The inclusion criteria were: (i) studies investigating bioactive compounds derived from agro-food by-products; (ii) studies reporting mechanistic, bioavailability, or clinical data; (iii) reviews providing comprehensive synthesis of relevant topics. Exclusion criteria were: (i) studies not related to food-derived bioactives; (ii) studies lacking sufficient methodological detail. This approach ensured a comprehensive and transparent selection of the literature discussed in this review.

Within the agri-food sector, the implementation of circular economy principles offers a dual benefit: mitigating the environmental impact of waste disposal and unlocking the intrinsic value of discarded biomass. The valorization of agri-food waste can take diverse pathways, from the production of bioenergy and bioplastics to the extraction of high-value bioactive compounds. For instance, the conversion of plant-based residues into biochar represents a green strategy that not only reduces waste but also enhances soil health and sequesters carbon, demonstrating a practical application of circularity within the supply chain [6]. However, the transition from a linear to a circular model is hindered by several interconnected barriers, including inadequate collection and sorting infrastructure, high processing costs, the lack of stable markets for recovered products, and a complex and often inconsistent regulatory landscape [2]. Despite these challenges, innovative technologies are rapidly evolving to overcome these hurdles, promising to make the bioeconomy a cornerstone of future food systems [7]. Emerging biotechnological innovations, including enzyme-assisted extraction, fermentation, and microbial bioconversion, are further expanding the possibilities for converting complex waste matrices into high-value products, enhancing both the efficiency and the sustainability of the valorization process [8,9].

### 1.2. Agro-Food Waste as a Reservoir of Bioactive Compounds

Agricultural and industrial processing of fruits, vegetables, and cereals generates enormous quantities of residues, including peels, seeds, pomace, husks, and pulps. These materials, once relegated to landfills or animal feed, are increasingly recognized as a rich and largely untapped source of bioactive phytochemicals. A growing body of evidence has established that these residues contain a remarkable diversity of bioactive compounds, including polyphenols, flavonoids, carotenoids, terpenes, and dietary fibers, whose concentrations can even exceed those found in the edible portions of the plant [10]. The composition and yield of these compounds are highly variable, depending on the plant species, cultivar, growing conditions, and, crucially, the extraction and processing methods employed [3].

Extensive non-clinical studies have demonstrated that these agro-industrial by-products exhibit a wide spectrum of biological activities, including potent antioxidant, anti-inflammatory, antimicrobial, and antiproliferative effects [11,12]. These therapeutic properties are largely attributed to their ability to modulate key cellular signaling pathways, offering potential strategies for the prevention and management of chronic non-communicable diseases. For example, the Mediterranean agri-food system, with its significant production of olives, grapes, and citrus fruits, generates a substantial amount of pomace, seeds, and peels that are particularly rich in nutraceutical molecules, making them a promising and sustainable source for the development of health-promoting products [13]. The potential of these biomasses extends beyond the traditional Mediterranean crops, as studies on tropical and subtropical residues, such as pineapple and Brazilian agro-food wastes, have also revealed significant concentrations of biofunctional compounds with promising health benefits [14,15]. The valorization of this biomass therefore represents a pivotal link between environmental sustainability and human health promotion.

### 1.3. Limits of Current Nutraceutical Strategies

Despite the immense potential of food-derived bioactive compounds as preventive and therapeutic agents, their translation into effective nutraceutical products faces significant biological, technological, and clinical challenges. A primary hurdle is their generally poor bioavailability, a result of low aqueous solubility, limited intestinal permeability, rapid metabolism in the gastrointestinal tract, and extensive first-pass hepatic metabolism [16]. Consequently, many of these promising compounds fail to reach target tissues in sufficient concentrations to exert their pharmacological effects. Furthermore, conventional nutraceutical formulations are often plagued by instability, subject to degradation by light, oxygen, and heat, which drastically reduces their shelf-life and efficacy.

Beyond bioavailability, the path from the laboratory to the clinic is fraught with obstacles. The inherent variability in the chemical composition of raw plant materials, influenced by genetic, agronomic, and environmental factors, makes it difficult to produce standardized extracts with consistent and reproducible biological activity [3]. This lack of standardization, coupled with limited knowledge of optimal dosage regimens and potential herb-drug interactions, impedes the design of robust clinical trials and undermines the credibility of nutraceuticals within the medical community. It is now well established that chronic diseases, which are the primary target for many nutraceuticals, require chronic intervention strategies, and the safety and efficacy of natural products over prolonged periods of use remain insufficiently characterized [17].

Therefore, the scientific community is tasked with not only identifying new bioactive molecules from waste sources but also developing advanced delivery systems, robust standardization protocols, and rigorous clinical evidence to bridge the gap between preclinical promise and tangible human health benefits [16].

## 2. Major Agro-Food Sources

The valorization of agro-food by-products begins with a critical assessment of the most abundant and promising residual biomasses generated by the global food industry. Rather than providing an exhaustive inventory, this section focuses on a selection of paradigmatic examples, citrus, olive, grape, tomato, and pomegranate residues, that exemplify the transition from waste to resource. Each of these sources is characterized by distinctive phytochemical profiles and has been the subject of extensive investigation regarding their biological activities and potential applications. Their selection is justified not only by their quantitative relevance in terms of generated waste volumes but also by the established scientific evidence supporting their nutraceutical value.

The five by-product sources selected—citrus, olive, grape, tomato, and pomegranate—were chosen based on three criteria: (i) their quantitative relevance in terms of global waste volumes, (ii) the extensive body of scientific evidence supporting their phytochemical richness and biological activities, and (iii) their representation of distinct phytochemical profiles and geographic origins, allowing a comprehensive overview of the field. Specifically, these five sources were selected because they represent distinct botanical families and phytochemical profiles (flavonoid-dominant in citrus, phenolic-rich in olive, anthocyanin-rich in grape, carotenoid-rich in tomato, and ellagitannin-rich in pomegranate), and because they are supported by a substantial body of the peer-reviewed literature documenting both their chemical characterization and biological activities.

The integration of green recovery technologies with chemical profiling has enabled a more comprehensive characterization of these matrices, facilitating the identification of their most valuable bioactive components and guiding their sustainable valorization [4]. The nutritional value and health effects of agri-food by-products have been systematically reviewed, highlighting their potential as sources of bioactive compounds for the development of functional foods and nutraceuticals [18].

Table 1 provides a synthetic overview of the main agro-food by-products discussed in this section, summarizing their major bioactive constituents and the principal biological activities documented in the literature.

While this review focuses on five paradigmatic sources, it is important to acknowledge that other agro-food by-products, including apple pomace, banana peel, coffee silverskin, and cereal bran, also represent valuable sources of bioactive compounds and have been the subject of extensive research [37,38]. Their exclusion from detailed discussion reflects a need for focus rather than a judgment on their scientific or commercial relevance.

The subsequent subsections will examine each of these sources in greater detail, highlighting the specific extraction strategies, phytochemical profiles, and health-promoting applications that have been investigated.

### 2.1. Citrus By-Products

The global citrus processing industry generates millions of tons of peel, pulp, and seed residues annually, representing up to 50% of the total fruit weight. These by-products are recognized as a particularly rich source of flavonoids, especially flavanones such as hesperidin, naringin, and eriocitrin, alongside polymethoxyflavones and hydroxycinnamic acids. The recovery of these bioactive compounds from citrus waste has been the subject of intense investigation, with significant advances in both conventional and innovative extraction approaches aimed at maximizing yield while preserving biological activity [19]. Among citrus species, bergamot (*Citrus bergamia*) has attracted particular attention due to the unique phytochemical composition of its peel and juice by-products. The valorization of bergamot residues through sustainable green extraction methods has yielded extracts rich in polyphenols with promising biological properties, including cardioprotective and metabolic benefits, which have been explored in both food applications and nutraceutical formulations [20].

The bioavailability of citrus flavonoids remains a challenge, yet strategies employing advanced delivery systems are under development to overcome these limitations and fully exploit the potential health effects of these widely available biomasses. Comprehensive strategies for the valorization of citrus by-products emphasize the importance of selecting appropriate extraction techniques tailored to the specific phytochemical profile of each residue, as well as the need to consider the geographical origin and processing history of the raw material [21].

### 2.2. Olive Pomace

Olive oil production is a cornerstone of the Mediterranean agri-food system, generating substantial quantities of pomace, a solid residue composed of pulp, skin, and stone fragments, alongside wastewater. Olive pomace is rich in phenolic compounds, particularly hydroxytyrosol, tyrosol, and oleuropein derivatives, which have documented antioxidant and anti-inflammatory properties. The nutraceutical potential of olive pomace has been extensively investigated, with cell-based studies demonstrating its ability to modulate oxidative stress and inflammatory pathways, while clinical studies have provided preliminary evidence of its beneficial effects on cardiovascular risk factors and metabolic health [24]. The valorization of this by-product not only addresses the environmental burden of olive oil production but also offers a sustainable source of bioactive molecules for the development of functional foods and supplements, contributing to the circular bioeconomy of Mediterranean regions [13].

### 2.3. Grape Pomace

The wine industry produces grape pomace, consisting of skins, seeds, and stems, as its primary solid waste, accounting for approximately 20–25% of the processed grape weight. This residue is a source of polyphenols, including anthocyanins, flavan-3-ols, proanthocyanidins, and stilbenes such as resveratrol, whose concentrations are often higher in the pomace than in the final wine product. The phenolic composition of grape pomace is well characterized, and its bioactivity has been demonstrated across multiple experimental models, showing antioxidant, anti-inflammatory, cardioprotective, and neuroprotective properties [25]. Beyond these biological effects, grape pomace has been explored for various applications, including the enrichment of food products with bioactive compounds and the development of nutraceutical formulations [26]. The valorization of this by-product represents a significant opportunity to enhance the sustainability of the wine industry while generating high-value ingredients for the health and food sectors. The phenolic composition of grape pomace has been extensively characterized, with studies demonstrating that the extraction conditions significantly influence the yield and bioactivity of the recovered compounds, highlighting the need for optimized protocols to maximize the nutraceutical potential of this residue [27]. A comprehensive review of grape pomace has confirmed the presence of a wide range of phenolic compounds with documented health benefits, including antioxidant, anti-inflammatory, and cardioprotective effects, and has highlighted the potential of this residue as a sustainable source of functional ingredients for food and nutraceutical applications [28].

### 2.4. Tomato By-Products

Tomato processing for sauces, pastes, and juices generates substantial quantities of pomace, consisting of skins, seeds, and fibrous material. This residue is particularly valued for its high content of carotenoids, especially lycopene, which is responsible for the characteristic red color and well-documented antioxidant activity. Additionally, tomato by-products contain significant levels of phenolic compounds, including chlorogenic acid, caffeic acid, and flavonoids, which contribute to the overall bioactivity of the extract. Recent strategies for the revalorization of tomato production and processing by-products have focused on the development of sustainable extraction methods to recover these bioactive compounds and on the evaluation of their potential applications in the food, pharmaceutical, and cosmetic industries [31]. The integration of tomato waste into circular economy models offers a dual advantage: reducing environmental pollution while providing a cost-effective source of health-promoting phytochemicals.

### 2.5. Pomegranate Residues

Pomegranate (*Punica granatum*) processing generates a significant amount of peel waste, which constitutes approximately 30–40% of the total fruit weight. Despite being discarded, pomegranate peel is rich in bioactive compounds, particularly ellagitannins, punicalagins, and ellagic acid, which are responsible for its documented antioxidant, antimicrobial, and anti-inflammatory activities. The potential health effects of pomegranate peel have been explored across various disease models, including cardiovascular disease, cancer, and metabolic disorders, with studies highlighting its ability to modulate key signaling pathways involved in oxidative stress and inflammation [33].

Recent studies have specifically explored the potential of pomegranate-derived bioactives in the management of metabolic dysfunction-associated steatotic liver disease and related comorbidities, highlighting their capacity to modulate lipid metabolism and reduce hepatic inflammation [39]. Despite this promising evidence, the translation of pomegranate peel extracts into effective nutraceutical products faces challenges related to standardization, bioavailability, and the need for rigorous clinical validation, issues that are common to many agro-food by-products and will be discussed in detail in subsequent sections. Pomegranate peel is recognized as a source of diverse bioactive compounds, including ellagitannins, flavonoids, and organic acids, whose physiological functions include antioxidant, anti-inflammatory, and antimicrobial activities that contribute to the prevention of chronic diseases [36]. Recent reviews have systematically catalogued the phytochemical composition of pomegranate peel, confirming the presence of ellagitannins, punicalagins, and ellagic acid as the major bioactive constituents, and have highlighted the influence of extraction methods on the yield and biological activity of the recovered compounds [34,35].

## 3. Bioactive Molecules

The valorization of agro-food by-products is fundamentally grounded in their notable chemical diversity. These residues harbor a complex array of bioactive phytochemicals, belonging to distinct chemical classes, each characterized by specific structural features and biological properties. Rather than providing an exhaustive catalog, this section focuses on the major classes of compounds that have been most extensively studied and that underpin the nutraceutical potential of the selected agro-food sources. The chemical heterogeneity of these biomasses, which includes polyphenols, flavonoids, terpenes, carotenoids, fatty acids, and peptides, is responsible for the pleiotropic biological activities observed in preclinical studies and offers multiple molecular targets for health promotion.

Recent advances in green extraction techniques have enabled the recovery of a wide range of bioactive compounds from plant-based residues, with studies on twenty different crops revealing significant variations in the phytochemical profiles of seeds, pomace, and peels, and emphasizing the importance of species-specific extraction strategies [37]. The systematic valorization of by-products from agri-food supply chains requires an integrated approach that considers the entire production process, from raw material selection to final product development, to ensure the sustainable recovery of functional ingredients with documented health benefits [5,40].

### 3.1. Polyphenols

Polyphenols represent one of the largest and most structurally diverse classes of bioactive compounds found in the plant kingdom, characterized by the presence of multiple phenolic hydroxyl groups attached to aromatic rings. These secondary metabolites are abundantly present in agro-food by-products, where they accumulate as a defense mechanism against environmental stressors. The polyphenolic profile of a given residue is highly specific to the plant species, tissue type, and processing conditions, contributing to the unique bioactivity of each matrix. The health-promoting properties of polyphenols, including their well-established antioxidant capacity and ability to modulate inflammatory pathways, have been extensively documented across various experimental models [3]. However, it is important to distinguish between antioxidant activity measured in chemical assays (e.g., DPPH, FRAP, ORAC) and antioxidant effects demonstrated in humans. Chemical assays provide a measure of radical-scavenging capacity in vitro, but they do not necessarily predict biological efficacy in vivo, where factors such as bioavailability, metabolism, and tissue distribution determine the actual antioxidant impact. Human studies using validated biomarkers of oxidative stress (e.g., F2-isoprostanes, oxidized LDL) are required to establish antioxidant effects in vivo.

The translation of these promising in vitro effects into clinical benefits is often hindered by the poor bioavailability and extensive metabolism of these compounds, which limits their systemic exposure and tissue distribution [16].

### 3.2. Flavonoids

Flavonoids constitute the most abundant subclass of polyphenols and are characterized by a common C6-C3-C6 carbon skeleton. Within agro-food residues, flavonoids are particularly concentrated in the peels, seeds, and pomace of fruits, where they contribute to color, flavor, and resistance to pathogens. The chemical diversity within this class is substantial, encompassing flavanones, flavones, flavonols, anthocyanins, and isoflavones, each with distinct biological activities and molecular targets. Among the most extensively studied sources, citrus by-products are rich in flavanones such as hesperidin, naringin, and eriocitrin, compounds that have been associated with cardioprotective and metabolic benefits [22]. The peel of citrus fruits also contains polymethoxyflavones, a specific subclass with enhanced lipophilicity and documented anti-inflammatory, antiproliferative (in preclinical models), and neuroprotective properties, which have attracted considerable interest for nutraceutical development [23]. The recovery and purification of these flavonoids from waste matrices, combined with strategies to improve their bioavailability, represent a major research focus for the sustainable production of functional ingredients.

A comprehensive understanding of flavonoid bioavailability, metabolic effects, and safety profiles is essential for the rational design of flavonoid-based nutraceutical interventions, as the biological activity of these compounds is profoundly influenced by their absorption, distribution, metabolism, and excretion characteristics [41,42].

### 3.3. Terpenes

Terpenes constitute a large and diverse class of naturally occurring hydrocarbons, built from isoprene units, that are widely distributed in plant tissues and often responsible for characteristic aromas. Agro-food by-products, particularly peels and pomace, retain significant amounts of volatile and non-volatile terpenoids, including mono- and sesquiterpenes, which are typically concentrated in the essential oil fraction. These compounds possess well-documented antimicrobial, antifungal, and antioxidant activities, making them valuable candidates for both food preservation and therapeutic applications [43]. The hydrophobic nature of many terpenes facilitates their interaction with microbial membranes, contributing to their biocidal activity, which has been explored as a natural alternative to synthetic preservatives [44]. Despite their promising bioactivity, the application of terpenes in nutraceutical and food products is constrained by their volatility, chemical instability, and limited aqueous solubility, necessitating the development of appropriate encapsulation and delivery strategies.

Beyond their antimicrobial properties, terpenes from agro-food by-products have been investigated for their anti-inflammatory, antioxidant, and anticancer activities. Monoterpenes such as limonene and perillyl alcohol, abundant in citrus peel essential oils, have demonstrated chemopreventive effects in preclinical models [45], while sesquiterpenes such as β-caryophyllene, found in various fruit and vegetable residues, act as selective agonists of the cannabinoid receptor type 2 (CB2) and exhibit anti-inflammatory activity [46]. The incorporation of these compounds into functional food formulations is limited by their volatility and susceptibility to oxidation, but encapsulation technologies, including spray drying and nanoemulsification, offer promising strategies to overcome these challenges [43].

### 3.4. Carotenoids

Carotenoids are a class of lipid-soluble pigments responsible for the yellow, orange, and red colors of many fruits and vegetables. These tetraterpenoids are characterized by a polyene chain that serves as the chromophore and confers their antioxidant properties, as well as their ability to quench singlet oxygen and scavenge free radicals. Agro-food by-products, particularly those derived from tomato processing, are a rich source of carotenoids, with lycopene representing the most abundant and extensively studied compound in these matrices [29]. Beyond lycopene, tomato residues contain significant amounts of β-carotene, lutein, and phytoene, each contributing to the overall bioactivity of the extract. The biological activities of carotenoids extend beyond antioxidant effects, encompassing modulation of cell signaling pathways involved in inflammation, cell proliferation, and apoptosis, which underpin their potential antiproliferative properties in preclinical models [30]. Evidence for cancer prevention in humans is currently lacking. In contrast, the poor aqueous solubility and limited oral bioavailability of carotenoids represent major challenges for their effective utilization, requiring formulation approaches that enhance their dissolution and intestinal absorption. The health benefits of lycopene and other carotenoids from tomato by-products have been investigated, with evidence from observational and some intervention studies suggesting a role in reducing the risk of chronic diseases, including cardiovascular disease and certain cancers, through mechanisms that extend beyond simple antioxidant activity [32].

### 3.5. Fatty Acids

Agro-food by-products, particularly seeds and pomace, retain significant quantities of lipids, including essential fatty acids with documented health benefits. These residues contain both saturated and unsaturated fatty acids, with the latter, particularly omega-3 and omega-6 polyunsaturated fatty acids, being of major interest for their anti-inflammatory and cardioprotective properties. The valorization of these lipid fractions can be achieved through fermentation processes, which enable the bioconversion of waste substrates into fatty acid-rich biomass [8]. The extraction and purification of fatty acids from agro-food residues offer a sustainable alternative to conventional sources, contributing to the circular bioeconomy while providing functional ingredients for food and nutraceutical applications. The incorporation of agri-food by-products into the dairy sector has been explored as a strategy to enhance the phytochemical content and functional properties of dairy products, with studies demonstrating the feasibility of enriching yogurts, cheeses, and fermented milks with bioactive compounds recovered from fruit and vegetable residues [47].

### 3.6. Peptides

Bioactive peptides are short amino acid sequences, typically consisting of 2–20 residues, that exert specific physiological effects beyond their nutritional value. These molecules can be released from parent proteins through enzymatic hydrolysis, fermentation, or gastrointestinal digestion, and their biological activities depend on their amino acid composition, sequence, and length. Agro-food by-products, including seeds, pomace, and press cakes, are increasingly recognized as promising sources of proteinaceous material that can be hydrolyzed to generate bioactive peptides with antioxidant, antihypertensive, antimicrobial, and immunomodulatory properties [48]. The recovery of these peptides from waste streams, combined with advances in purification and characterization techniques, represents an emerging frontier in the valorization of agro-food residues.

Although peptides are not the primary focus of this review, their presence and potential contribution to the bioactivity of agro-food extracts should not be overlooked. Phenolic compounds, which are among the most abundant bioactive constituents of agro-food by-products, have been the subject of extensive research regarding their bioavailability, metabolism, and health effects, with evidence indicating that their biological activity is modulated by interactions with the gut microbiota and by the formation of metabolites with distinct bioactivity profiles [49].

In addition to the major classes discussed above, agro-food by-products also contain minerals (e.g., potassium, magnesium, calcium) and vitamins (e.g., vitamin C, vitamin E, B-complex vitamins) that contribute to their nutritional value. While these micronutrients are generally present at lower concentrations than in the edible portions, their recovery from by-products can contribute to the development of nutrient-rich functional ingredients. However, their bioavailability and interaction with other phytochemicals remain insufficiently characterized and warrant further investigation.

### 3.7. Dietary Fibers and Polysaccharides

Dietary fibers and polysaccharides represent another important class of bioactive compounds derived from agro-food by-products, particularly from cereal brans, fruit peels, and pomaces. These compounds include pectin, cellulose, hemicellulose, β-glucans, and resistant starch, which exert their health effects primarily through modulation of the gut microbiota and improvement of intestinal function [10]. Unlike polyphenols, dietary fibers are not absorbed intact in the small intestine but are fermented by colonic bacteria to produce short-chain fatty acids (SCFAs), including acetate, propionate, and butyrate, which serve as energy substrates for colonocytes and modulate immune and metabolic functions. Clinical evidence for the beneficial effects of dietary fibers is substantial, with human studies demonstrating reductions in LDL cholesterol (particularly for β-glucans and pectin), improved glycemic control, and enhanced gut barrier integrity [10]. Citrus pomace is particularly rich in pectin, while cereal brans are important sources of β-glucans and arabinoxylans. The incorporation of these fiber-rich by-products into functional foods represents a well-established strategy for improving nutritional quality and promoting gut health.

## 4. Molecular Mechanisms

The health-promoting effects of bioactive compounds derived from agro-food by-products are ultimately mediated by their ability to modulate a complex network of intracellular signaling pathways. Rather than acting through a single molecular target, these phytochemicals exert pleiotropic effects, simultaneously influencing multiple pathways that govern cellular stress responses, inflammation, metabolism, and survival. Understanding these mechanisms is essential not only to validate the traditional uses of plant-derived remedies but also to identify the molecular basis for the development of evidence-based nutraceutical interventions. This section provides an overview of the principal signaling cascades targeted by agro-food bioactives, with particular emphasis on those most relevant to the prevention and management of chronic diseases. Throughout this section, evidence is presented with explicit reference to the experimental model employed (in vitro, animal, or human), and the strength of the evidence is qualified accordingly. It is also essential to distinguish between pathway modulation (observed changes in signaling intermediates) and demonstrated causal involvement (evidence that pathway inhibition or genetic ablation abolishes the observed effect), as well as between general autophagy and selective mitophagy, which are regulated by distinct molecular machinery and serve different cellular functions. The experimental evidence discussed below derives predominantly from cell culture and animal models, and the concentrations and doses employed should be considered when assessing translational relevance.

For each molecular mechanism discussed below, we specify the representative compound(s), the by-product source (where applicable), the experimental model, and the level of evidence (in vitro, animal, or human). Where compounds are not derived from the five selected by-products, they are explicitly identified as external mechanistic examples. Studies employing isolated compounds (e.g., resveratrol, curcumin, quercetin) cannot be directly extrapolated to the efficacy of complex by-product extracts, which contain multiple phytochemicals whose interactions may modulate biological activity; where such compounds are discussed, they serve as mechanistic examples rather than as constituents of the specific by-products covered in Section 2. Similarly, findings from cell culture and animal models must be distinguished from human evidence, and the experimental model, dose, and direction of effect should be considered when interpreting mechanistic data. It should also be noted that while review articles provide comprehensive synthesis, primary research studies remain essential for establishing causal relationships and for identifying novel mechanisms. Where possible, we have cited primary research; however, the reliance on reviews for some mechanistic pathways reflects the breadth of the topic and the need for synthesis.

Table 2 provides a synthetic overview of the principal molecular mechanisms modulated by agro-food-derived bioactive compounds, summarizing the key signaling pathways involved, their biological effects, and representative phytochemicals that act upon them. In Table 2, representative compounds are listed to illustrate pathway modulation; some (e.g., resveratrol, hydroxytyrosol, punicalagin) are endogenous to the by-products discussed in Section 2, whereas others (e.g., curcumin, quercetin) are external mechanistic examples.

The subsequent subsections will delve into each of these mechanisms in greater detail, examining the specific molecular interactions and the experimental evidence supporting the role of agro-food bioactives in modulating these pathways.

### 4.1. Nrf2 and the Antioxidant Response

The nuclear factor erythroid 2-related factor 2 (Nrf2) is a master transcription factor that orchestrates the cellular defense against oxidative stress. Under basal conditions, Nrf2 is sequestered in the cytoplasm by its inhibitor Keap1, which promotes its ubiquitination and proteasomal degradation. Upon exposure to electrophilic or oxidative stimuli, Nrf2 dissociates from Keap1, translocates to the nucleus, and binds to the antioxidant response element (ARE) in the promoter region of target genes. This transcriptional activation leads to the coordinated upregulation of a battery of cytoprotective enzymes, including heme oxygenase-1 (HO-1), NAD(P)H:quinone oxidoreductase 1 (NQO1), and glutathione S-transferases, which collectively enhance the cellular capacity to neutralize reactive oxygen species and detoxify xenobiotics [50]. The activation of the Nrf2 pathway by dietary phytochemicals, including flavonoids, polyphenols, and terpenoids, is a well-established mechanism underlying their antioxidant and chemopreventive properties [51]. Among the compounds derived from agro-food by-products, several have been identified as Nrf2 inducers, and their ability to activate this pathway has been linked to the preservation of mitochondrial integrity and the prevention of oxidative damage in various disease models [52]. The activation of Nrf2 by dietary phytochemicals is also linked to the regulation of lipid peroxidation, a process that plays a critical role in the pathogenesis of cancer and other oxidative stress-related diseases, highlighting the potential of Nrf2 modulators derived from agro-food sources [50]. The potential of Nrf2 modulation extends beyond antioxidant defense, as this transcription factor also regulates inflammatory responses, autophagy, and mitochondrial biogenesis, making it a central hub in the cellular stress response network.

### 4.2. NF-κB and Inflammatory Signaling

The nuclear factor kappa-light-chain-enhancer of activated B cells (NF-κB) is a family of transcription factors that plays a pivotal role in the regulation of immune responses, inflammation, and cell survival. In resting cells, NF-κB is sequestered in the cytoplasm through association with inhibitor of κB (IκB) proteins. In response to pro-inflammatory stimuli, including tumor necrosis factor-α (TNF-α), interleukin-1β (IL-1β), and lipopolysaccharide (LPS), IκB kinase (IKK) is activated, leading to the phosphorylation and subsequent proteasomal degradation of IκB. This process releases NF-κB, allowing its translocation to the nucleus, where it promotes the transcription of a wide array of pro-inflammatory genes, including cytokines, chemokines, adhesion molecules, and inducible enzymes such as cyclooxygenase-2 (COX-2) and inducible nitric oxide synthase (iNOS) [53]. Chronic activation of NF-κB is a hallmark of many inflammatory diseases and contributes to the pathogenesis of cancer, cardiovascular disease, and metabolic disorders. Agro-food-derived bioactives, particularly polyphenols and flavonoids, have been shown to exert anti-inflammatory effects by interfering with multiple steps of the NF-κB signaling cascade, including inhibition of IKK activity, prevention of IκB degradation, and suppression of NF-κB nuclear translocation [54]. This capacity to modulate NF-κB-dependent gene expression underpins the beneficial effects of these compounds in reducing chronic low-grade inflammation and mitigating the progression of inflammation-associated pathologies. Punicalagin, a bioactive compound from pomegranate, has been shown to regulate multiple signaling pathways involved in inflammation-associated chronic diseases, including NF-κB, suggesting its potential as a multitarget anti-inflammatory agent [55].

### 4.3. AMPK and SIRT1 in Metabolic Regulation

The AMP-activated protein kinase (AMPK) and sirtuin 1 (SIRT1) are two interconnected energy-sensing molecules that play a central role in the maintenance of cellular and organismal metabolic homeostasis. AMPK is activated by an increase in the cellular AMP/ATP ratio, a condition that signals energy depletion, and once activated, it phosphorylates downstream targets to promote catabolic pathways that generate ATP while inhibiting anabolic processes that consume energy [56]. SIRT1, an NAD^+^-dependent deacetylase, is activated under conditions of low energy status and, together with AMPK, orchestrates a coordinated adaptive response to metabolic stress. This AMPK-SIRT1 axis regulates multiple aspects of cellular metabolism, including glucose and lipid homeostasis, mitochondrial function, and insulin sensitivity, through the deacetylation and phosphorylation of key transcriptional regulators such as peroxisome proliferator-activated receptor gamma coactivator 1-alpha (PGC-1α) and forkhead box O (FoxO) transcription factors [51]. Bioactive compounds from agro-food sources, including resveratrol, curcumin, and various flavonoids, have been shown to activate this pathway, thereby improving mitochondrial function and enhancing cellular resilience to metabolic stress [53]. The activation of AMPK and SIRT1 by dietary phytochemicals has been implicated in the prevention of metabolic diseases, including obesity, type 2 diabetes, and metabolic dysfunction-associated steatotic liver disease (MASLD), through the restoration of metabolic flexibility and the reduction in ectopic lipid accumulation [56]. This pathway also intersects with autophagy and mitochondrial quality control, further amplifying its impact on cellular health.

### 4.4. Autophagy and Mitophagy

Autophagy is an evolutionarily conserved catabolic process that mediates the degradation and recycling of damaged organelles, misfolded proteins, and intracellular pathogens through the lysosomal pathway. This process is essential for the maintenance of cellular homeostasis, particularly under conditions of nutrient deprivation, oxidative stress, and organelle damage. A specialized form of autophagy, mitophagy, selectively targets damaged or dysfunctional mitochondria for elimination, thereby preserving mitochondrial quality and preventing the release of pro-apoptotic factors and reactive oxygen species [63]. The PINK1-Parkin pathway is a key regulator of mitophagy, wherein the accumulation of PINK1 on the outer mitochondrial membrane of depolarized mitochondria recruits Parkin, leading to the ubiquitination of mitochondrial proteins and the subsequent sequestration of the organelle into autophagosomes [52].

Extensive evidence has demonstrated that polyphenols derived from agro-food by-products, particularly resveratrol and hydroxytyrosol, can modulate mitophagy by activating the PINK1-Parkin pathway and enhancing the clearance of dysfunctional mitochondria [58,59]. Punicalagin, a major ellagitannin found in pomegranate peel, has been shown to modulate general autophagy signaling pathways in LPS-stimulated macrophages, specifically through inhibition of FoxO3a/autophagy signaling [57]. However, this study does not establish PINK1/Parkin-mediated mitophagy by punicalagin, and the findings should be interpreted as evidence for autophagy modulation rather than selective mitophagy, consistent with the distinction outlined above. This ability to promote mitochondrial quality control is emerging as a key mechanism underlying the cytoprotective and anti-aging effects of these compounds, and represents a promising target for interventions aimed at mitigating age-related mitochondrial dysfunction and metabolic decline.

### 4.5. Mitochondrial Homeostasis

Mitochondria are dynamic organelles that continuously undergo cycles of fission and fusion, a process that is essential for the maintenance of their morphology, distribution, and functional integrity. These mitochondrial dynamics, together with mitochondrial biogenesis and mitophagy, constitute a coordinated network that ensures the quality control and functional adaptation of the mitochondrial pool in response to cellular demands. Imbalances in this network, characterized by excessive fission, impaired fusion, or defective mitophagy, are associated with a range of pathological conditions, including metabolic syndrome, neurodegeneration, and cardiovascular disease [60]. Bioactive compounds from agro-food by-products have emerged as promising modulators of mitochondrial homeostasis. Hydroxytyrosol, a major phenolic component of olive pomace, has been shown to exert protective effects on mitochondrial function by regulating fusion/fission dynamics, promoting mitochondrial biogenesis, and enhancing the activity of the electron transport chain [60]. Similarly, other polyphenols and flavonoids can preserve mitochondrial integrity through the activation of the AMPK-SIRT1-PGC-1α axis, which stimulates the expression of nuclear-encoded mitochondrial genes and enhances oxidative phosphorylation capacity [51]. The ability of these compounds to maintain mitochondrial homeostasis is increasingly recognized as a central mechanism contributing to their beneficial effects on metabolic health, energy balance, and cellular resilience to stress. The role of phenolic compounds in modulating mitochondrial function has been further elucidated through studies demonstrating their ability to regulate the expression of genes involved in mitochondrial biogenesis and dynamics, as well as to protect mitochondrial DNA from oxidative damage [61].

### 4.6. Apoptosis

Apoptosis, or programmed cell death, is a tightly regulated physiological process that eliminates damaged, infected, or superfluous cells without inducing an inflammatory response. This process can be initiated through two principal pathways: the intrinsic (mitochondrial) pathway, which is triggered by intracellular stress signals, and the extrinsic (death receptor) pathway, which is activated by the binding of extracellular ligands to cell surface receptors. Both pathways converge on the activation of a cascade of cysteine-aspartic proteases (caspases) that orchestrate the execution phase of apoptosis [62]. The ability of natural products to modulate apoptotic pathways has attracted considerable interest, particularly in the context of cancer chemoprevention and therapy.

Agro-food-derived bioactives can induce apoptosis in cancer cells through the intrinsic pathway by disrupting mitochondrial membrane potential, promoting the release of cytochrome c, and activating caspase-9 and caspase-3, while also sensitizing cells to extrinsic death receptor signaling [62]. Conversely, in the context of degenerative diseases, these compounds can exert anti-apoptotic effects, protecting cells from stress-induced cell death through the upregulation of survival pathways such as PI3K/Akt and the inhibition of pro-apoptotic Bcl-2 family members [53]. The dual role of these phytochemicals as both pro-apoptotic and anti-apoptotic agents highlights their context-dependent actions, which are governed by the cellular environment, the nature of the stress signal, and the specific molecular targets engaged. This complexity underscores the need for a nuanced understanding of the molecular mechanisms underlying the biological activities of agro-food-derived bioactives to guide their safe and effective application in disease prevention and therapy.

## 5. The Translational Bottleneck

Despite the impressive body of preclinical evidence demonstrating the health-promoting potential of bioactive compounds derived from agro-food by-products, their translation into effective nutraceutical products for human use remains severely limited. This gap between laboratory findings and clinical applications is not merely a technical hurdle but a fundamental bottleneck that undermines the credibility and therapeutic utility of nutraceuticals. The challenges are multifaceted, spanning from the intrinsic properties of the bioactive molecules themselves to the variability of raw materials, the lack of standardized protocols, and the profound differences between simplified in vitro systems and the complex physiology of the human organism. This section critically examines the principal obstacles that impede the successful translation of agro-food-derived bioactives into evidence-based interventions, providing a framework for understanding why many promising compounds fail to deliver their expected benefits in clinical settings.

To critically assess the current state of clinical evidence, Table 3 summarizes the principal human intervention studies conducted with preparations derived from the five agro-food by-products discussed in this review. This table includes information on the preparation and its provenance, study design, population, dose, duration, comparator, main outcomes, adverse events, and key limitations. Several important observations emerge from this synthesis. First, while a growing number of clinical trials have been conducted with by-product-derived preparations, most studies are small (typically fewer than 100 participants), of short duration (ranging from 2 weeks to 6 months), and often employ heterogeneous preparations that are not directly comparable [64]. Second, the clinical evidence is unevenly distributed across the five by-product sources: olive pomace and grape pomace have been the subject of multiple randomized controlled trials, whereas citrus by-products have been investigated primarily through dietary interventions rather than isolated preparations, and direct human evidence for pomegranate peel remains limited. Third, while several studies have demonstrated statistically significant improvements in intermediate endpoints, such as reductions in oxidized LDL, inflammatory markers (hs-CRP, IL-6, MCP-1, NF-κB), and improvements in insulin sensitivity and visual outcomes, none have evaluated hard clinical endpoints such as cardiovascular events, cancer incidence, or mortality [65]. Fourth, the interpretation of these findings is complicated by the use of different preparations (biscuits, tablets, beverages, extracts), varying doses of bioactive compounds, and the absence of standardized chemical characterization in many studies. This issue is further compounded by the intrinsically low oral bioavailability of many citrus flavonoids and other polyphenols, which substantially impairs their in vivo efficacy and contributes to the difficulty of translating preclinical findings into clinical benefits [66]. Finally, adverse events have been poorly reported across most trials, limiting the assessment of tolerability and safety. These observations underscore the need for larger, longer-term, well-designed clinical trials with standardized preparations and clinically meaningful endpoints to establish the true health-promoting potential of agro-food by-product-derived nutraceuticals.

A decade of research has established that the recovery of nutrients and bioactive compounds from agri-food by-products has progressed significantly, yet the translation of these findings into clinically effective nutraceuticals remains limited by the persistent challenges of bioavailability, standardization, and the need for robust clinical validation [5].

### 5.1. Standardization of Extracts

The lack of robust standardization protocols represents one of the most critical barriers to the clinical translation of nutraceuticals. Unlike synthetic pharmaceuticals, which consist of a single, well-defined active ingredient with known purity and potency, plant-derived extracts are complex mixtures of hundreds of chemically distinct compounds, whose composition varies considerably depending on the plant species, cultivar, geographical origin, harvesting time, and extraction method. The chemical characterization of these extracts requires a comprehensive approach that integrates multiple analytical techniques, including chromatographic fingerprinting, spectroscopic profiling, and quantitative analysis of marker compounds [76]. Best practice guidelines emphasize the need for a detailed description of the botanical identity, growth conditions, and processing steps, coupled with a thorough phytochemical profiling to ensure batch-to-batch consistency [77]. The quantification of specific bioactive markers, using techniques such as high-performance thin-layer chromatography (HPTLC), high-performance liquid chromatography (HPLC), and mass spectrometry, is essential to establish quality control parameters and to correlate the chemical composition with biological activity [78]. The emerging application of metabolomics to the standardization of herbal extracts offers a powerful approach to capture the full chemical complexity of these matrices and to identify quality markers that are robust to environmental and processing variability [79]. Without such quality assessment, the reproducibility of preclinical and clinical studies is compromised, and the interpretation of results across different studies becomes virtually impossible. The complexity of botanical extracts necessitates the integration of multiple analytical platforms, including chromatographic, spectroscopic, and biological assays, to ensure comprehensive quality control and to establish meaningful correlations between chemical composition and biological activity [80].

### 5.2. Variability of Raw Materials

The chemical composition of plant materials is inherently variable, influenced by a complex interplay of genetic, agronomic, and environmental factors that are largely beyond the control of the manufacturer. Genetic diversity among cultivars, differences in soil composition and nutrient availability, climatic conditions, seasonal variations, and post-harvest handling practices all contribute to the phytochemical heterogeneity of the raw material [81]. This variability is particularly pronounced in agro-food by-products, which are derived from diverse production chains and are subject to fluctuations in quality depending on the primary processing operations. A striking example of this issue is provided by the analysis of commercial quercetin supplements, which revealed that the actual quercetin content ranged from 14.8% to 106.7% of the label claim, demonstrating the dramatic discrepancies between declared and actual composition [82]. This variability not only affects the reproducibility of biological effects but also poses significant challenges for the design of clinical trials, where consistent dosing and exposure are essential for the interpretation of outcomes. The establishment of traceability systems and the implementation of good agricultural and collection practices are critical to mitigate this source of uncertainty, yet they are not widely adopted in the nutraceutical industry.

Safety considerations specific to recovered by-products warrant particular attention. These include the potential presence of environmental contaminants (heavy metals, pesticides, mycotoxins), residual solvents from extraction processes, and the inherent batch-to-batch variability that complicates the establishment of safe upper limits. Tolerability at proposed doses, particularly for long-term consumption, remains insufficiently characterized for many by-product-derived preparations. Additionally, claims regarding sustainability, cost-effectiveness, and scalability should be qualified, as supporting process-level evidence, including life-cycle assessments and industrial-scale feasibility studies, is often limited or unavailable.

The fermentation of agro-food by-products has emerged as a promising strategy to enhance the nutritional value and bioactivity of these matrices, while also contributing to the reduction in waste and the production of functional ingredients with improved standardization potential [9].

### 5.3. Bioavailability

Even when standardized extracts are produced with consistent composition, the biological activity of agro-food-derived bioactives is severely constrained by their limited absorption and rapid metabolism. The term bioavailability encompasses the fraction of an ingested compound that is released from the food matrix, absorbed through the intestinal epithelium, and reaches the systemic circulation in an active form. Systematic analyses of polyphenol bioavailability, based on a comprehensive review of 97 human studies, have demonstrated that the plasma concentrations of these compounds are typically in the low micromolar to nanomolar range, far below the concentrations required to elicit the biological effects observed in cell culture experiments [83].

For example, pharmacokinetic studies in humans have shown that the plasma Cmax of hydroxytyrosol after consumption of 5 mg in extra virgin olive oil is approximately 24.6 nmol/L at 30 min (Tmax), while the Cmax of curcumin from unformulated turmeric extract is typically below 5 ng/mL, with a relative bioavailability that can be increased up to 39-fold by cyclodextrin-based formulations [84]. Similarly, the Cmax of punicalagin metabolites (urolithins) in human plasma after pomegranate consumption ranges from 0.5 to 2 μmol/L, depending on the individual’s gut microbiota metabotype [85].

However, it is important to avoid the implication that low plasma concentrations of parent compounds necessarily exclude biological effects. Metabolites generated in the intestinal lumen or by the gut microbiota may reach higher local concentrations in the gastrointestinal tract and liver, and may themselves possess biological activity distinct from the parent compound. Bioaccessibility (the fraction released from the food matrix and available for absorption), absorption (the fraction crossing the intestinal epithelium), systemic exposure (circulating concentrations of parent compounds and metabolites), and biological activity at target tissues are distinct parameters that should not be conflated.

It is also important to distinguish between the concentration of bioactive compounds in an extract and their bioavailability. Increasing the concentration does not necessarily translate into increased physiological exposure, because absorption and metabolism are saturable processes. Therefore, formulation strategies aimed at enhancing bioavailability are often more effective than simply increasing the dose of a poorly bioavailable compound.

The assessment of these parameters requires complementary methodological approaches. In vitro gastrointestinal digestion models simulate the release of compounds from the food matrix (bioaccessibility), while Caco-2 cell monolayers provide estimates of intestinal transport and absorption [4]. Pharmacokinetic studies in humans or animals quantify systemic exposure (Cmax, Tmax, AUC), and metabolomic analyses of plasma and urine enable the identification and quantification of circulating metabolites, including those derived from gut microbiota [11]. The integration of these approaches provides a comprehensive picture of the fate of bioactive compounds after ingestion.

The factors limiting bioavailability include low aqueous solubility, poor intestinal permeability, active efflux by transporters such as P-glycoprotein, and extensive first-pass metabolism by phase I and phase II enzymes in the intestine and liver. The recovery of polyphenols from agro-food by-products through membrane-based processes, such as nanofiltration and tight ultrafiltration, can yield purified fractions with enhanced concentration, but this does not address the fundamental physiological barriers to absorption [86]. Nanotechnology-based delivery systems, including lipid nanoparticles, polymeric nanoparticles, and nanoemulsions, have been developed to improve the solubility, stability, and intestinal absorption of poorly bioavailable nutraceuticals, yet their clinical application remains limited [16]. In a preclinical study conducted in rats, waste-based microencapsulation has been explored to improve the bioavailability of bioactive compounds from *Citrus bergamia* by-products, demonstrating the potential of innovative delivery systems to enhance systemic exposure in an animal model [87]. Whether these findings translate to human bioavailability remains to be established. The bioavailability of dietary flavonoids is influenced by multiple factors, including their chemical structure, the food matrix, and the metabolic activity of the gut microbiota, which together determine the systemic exposure to both parent compounds and their bioactive metabolites [42].

### 5.4. Intestinal Metabolism

The bioavailability of dietary bioactives is further complicated by their extensive metabolism within the gastrointestinal tract, mediated by both host enzymes and the gut microbiota. The intestinal epithelium expresses a range of conjugating enzymes, including UDP-glucuronosyltransferases and sulfotransferases, which rapidly convert absorbed aglycones into glucuronide and sulfate conjugates that are more hydrophilic and more readily excreted, but often less biologically active than their parent compounds. More importantly, the gut microbiota, a complex ecosystem of trillions of microorganisms, plays a central role in the metabolism of polyphenols and other phytochemicals [88]. These microbial communities perform a diverse array of biotransformations, including deglycosylation, ring cleavage, dehydroxylation, and demethylation, generating metabolites that differ substantially from the parent compounds in terms of their chemical structure and biological activity. The microbial metabolism of proanthocyanidins, for example, yields low-molecular-weight phenolic acids and aromatic catabolites that are absorbed and may contribute to the systemic effects attributed to the parent oligomers [88,89]. Importantly, the composition and metabolic capacity of the gut microbiota are highly variable among individuals, influenced by diet, age, genetics, and health status, leading to inter-individual differences in the generation of bioactive metabolites and, consequently, in the response to nutraceutical interventions. The gut microbiota plays a dual role in the metabolism of dietary bioactives, as microbial enzymes can both activate and deactivate phytochemicals, generating metabolites with distinct biological activities that may contribute to the observed health effects of polyphenol-rich diets [90]. This person-to-person variability represents a major challenge for the development of universally effective nutraceutical products.

### 5.5. Effective Dose

The determination of the optimal effective dose for nutraceutical interventions is fraught with uncertainty, as the relationship between the administered dose, the circulating concentrations of active compounds and metabolites, and the biological response is often nonlinear and context-dependent. The lack of systematic dose-finding studies, combined with the absence of validated biomarkers of exposure and response, hampers the rational design of clinical trials and contributes to the inconsistent results observed in the nutraceutical literature. This uncertainty is further compounded by the gap between in vitro testing conditions and human physiology.

In cell culture experiments, compounds are typically tested at concentrations ranging from micromolar to millimolar, which are readily achievable in the artificial environment of the culture dish. However, these concentrations are seldom attained in human plasma following oral administration, even at high dietary intakes, due to the bioavailability limitations discussed above. The translation of in vitro potency data to human dosing regimens is therefore fraught with error, and many clinical trials have failed to demonstrate efficacy because the dose selected was insufficient to achieve biologically relevant concentrations at the target site [91]. Furthermore, the dose–response relationship for many phytochemicals is not monotonic but may exhibit U-shaped or biphasic patterns, where low doses are protective and high doses are harmful or ineffective, a phenomenon known as hormesis.

Pharmacokinetic parameters such as Cmax, Tmax, and AUC are essential for understanding the relationship between dose and systemic exposure. For representative compounds, these parameters have been characterized in human studies; for example, the Cmax of hydroxytyrosol after consumption of 5 mg in extra virgin olive oil is approximately 24.6 nmol/L at 30 min, while the Cmax of curcumin from unformulated turmeric extract is typically below 5 ng/mL [92,93]. These quantitative data highlight the importance of considering pharmacokinetics in the design of nutraceutical interventions and in the interpretation of dose–response relationships.

The emerging field of nutrigenomics, which integrates genetic, metabolic, and microbiome data, offers a framework for understanding the inter-individual variability in response to nutraceutical interventions and for identifying biomarkers that can guide dose optimization and personalized recommendations [94].

### 5.6. Gap Between Cellular Models and Humans

Perhaps the most fundamental challenge in the translation of agro-food-derived bioactives is the profound gap between the simplified cellular and animal models used in preclinical research and the complex physiology of the human organism. Cell culture models, while invaluable for mechanistic studies, are reductionist systems that lack the three-dimensional tissue architecture, the dynamic intercellular communication, and the systemic regulatory networks that characterize the in vivo environment. The concentrations of compounds used in these models, the absence of plasma protein binding, and the lack of metabolic transformation by the liver and gut microbiota all contribute to an overestimation of biological activity that rarely translates to humans. Animal models, despite their greater physiological complexity, also have limitations, including differences in drug metabolism, transport, and target sensitivity, as well as the use of standardized diets and controlled environments that do not reflect the variability of human lifestyles and exposures [95]. The conduct of well-designed clinical trials, with appropriate dosing, adequate sample sizes, and robust endpoints, is essential to bridge this gap, yet such studies are costly and complex, and they remain the exception rather than the norm in the nutraceutical field. The use of validated biomarkers of exposure, such as the quantification of specific metabolites in plasma and urine, along with the application of omics technologies to capture the individual response to intervention, may help to refine clinical trial design and to identify those individuals most likely to benefit from nutraceutical strategies. The establishment of robust regulatory frameworks and the implementation of nutrivigilance systems are essential to ensure the safety and efficacy of nutraceutical products, while also fostering innovation and consumer trust in the sector [92]. Only through the rigorous integration of preclinical and clinical research can the translational gap be overcome and the full therapeutic potential of agro-food-derived bioactives be realized.

**Figure 1:**
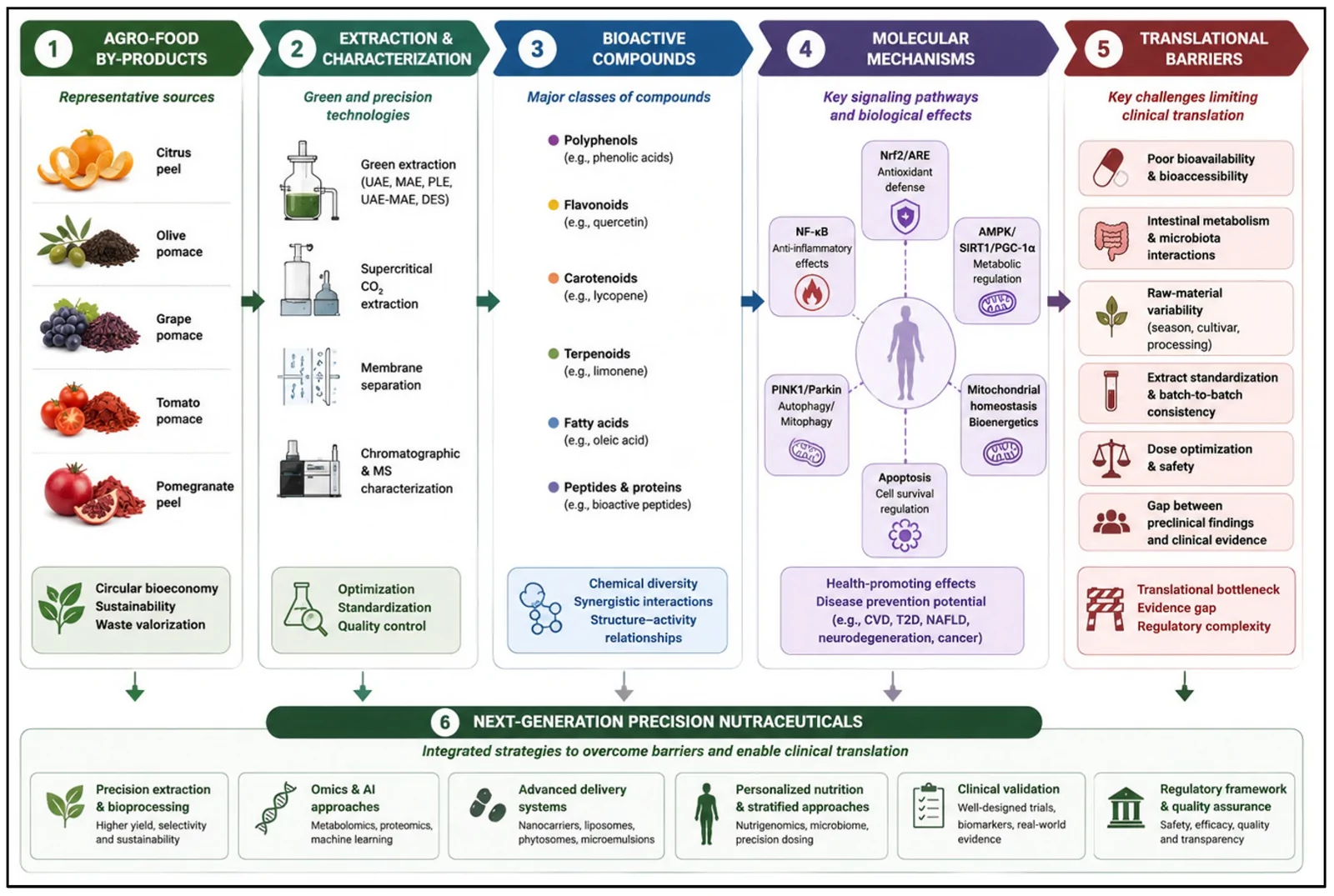
From agro-food by-products to precision nutraceuticals: an integrated translational framework. Representative agro-food residues (citrus, olive, grape, tomato, and pomegranate) serve as sources of diverse bioactive compounds, including polyphenols, flavonoids, carotenoids, terpenoids, fatty acids, and peptides. Following green and precision extraction and chemical characterization, these compounds can modulate key molecular pathways involved in oxidative stress, inflammation, metabolic regulation, mitochondrial homeostasis, autophagy/mitophagy, and apoptosis. Their translation into clinically meaningful nutraceutical interventions is limited by raw-material variability, extract standardization, poor bioavailability, intestinal metabolism, dose uncertainty, and the gap between preclinical and clinical evidence. Precision extraction, advanced delivery systems, omics and artificial intelligence, personalized nutrition, clinical validation, and appropriate regulatory frameworks represent interconnected strategies to overcome these barriers and enable the development of sustainable, safe, and effective nutraceutical products.

The overall translational pathway from agro-food by-products to precision nutraceuticals is summarized in Figure 1. This framework highlights the interconnected steps linking the recovery and characterization of bioactive compounds to their molecular effects, while emphasizing the biological, technological, and regulatory barriers that currently limit clinical translation. It also illustrates how precision extraction, advanced delivery systems, omics-based approaches, artificial intelligence, personalized nutrition, and clinical validation may contribute to overcoming these limitations.

## 6. Future Perspectives

The translation of agro-food by-products into effective nutraceutical interventions, while fraught with challenges, also presents unprecedented opportunities for innovation across multiple domains. The convergence of advances in extraction technologies, systems biology, personalized nutrition, and regulatory science is poised to transform the current landscape, enabling the development of evidence-based, sustainable, and individualized health-promoting strategies. This section outlines the most promising directions for future research and development, highlighting how emerging technologies and interdisciplinary approaches can address the translational bottlenecks identified in the previous section.

### 6.1. Precision Extraction

The evolution of extraction technologies towards greater selectivity, efficiency, and sustainability represents a cornerstone of future nutraceutical development. Conventional extraction methods, while widely used, are often nonspecific, energy-intensive, and reliant on toxic organic solvents, resulting in extracts with variable composition and potential environmental burden. The advent of green extraction technologies, including ultrasound-assisted extraction, microwave-assisted extraction, pressurized liquid extraction, and supercritical fluid extraction, has significantly improved the recovery of bioactive compounds while reducing solvent consumption and processing time [93]. However, the next frontier lies in the concept of precision extraction, wherein extraction processes are designed not merely to maximize yield but to selectively enrich specific bioactive fractions or to preserve the structural integrity and bioactivity of labile compounds. Deep eutectic solvents (DESs) and their natural counterparts (NADESs) have emerged as particularly promising tools for this purpose, as they can be tailored to interact with specific classes of phytochemicals through hydrogen bonding, van der Waals forces, and other non-covalent interactions, thereby enhancing both extraction efficiency and the biological activity of the recovered compounds [96]. The integration of machine learning and artificial intelligence into extraction process optimization further enhances the precision of these approaches, enabling the prediction of optimal solvent composition and processing parameters based on the chemical characteristics of the target matrix. This paradigm shift towards precision extraction may enable the production of extracts with consistent composition, enhanced bioactivity, and reduced environmental footprint, laying the foundation for the industrial-scale production of high-quality nutraceutical ingredients. The characterization and correct use of eutectic solvents require standardized guidelines to ensure the reproducibility of extraction processes and the comparability of results across studies, which are essential prerequisites for the industrial adoption of these innovative solvents [97]. The opportunities and challenges associated with deep eutectic liquids as tailorable extraction solvents have been systematically reviewed, highlighting their potential for selective extraction of bioactive compounds from complex plant matrices [98].

### 6.2. Omics Technologies

The application of omics technologies, genomics, transcriptomics, proteomics, metabolomics, and microbiomics, is advancing our understanding of the interactions between dietary bioactives and human physiology. These high-throughput approaches enable the comprehensive profiling of the molecular responses to nutraceutical interventions, providing insights into the mechanisms of action, the identification of biomarkers of exposure and response, and the characterization of individual variability in metabolism and efficacy [99]. The integration of multi-omics data, combined with advanced bioinformatics and systems biology approaches, allows the construction of detailed molecular networks that map the relationships between phytochemical exposure, metabolic transformation, and biological outcomes. This systems-level perspective is particularly valuable for understanding the pleiotropic effects of agro-food-derived bioactives, which act on multiple molecular targets and pathways simultaneously. The application of computational and AI-assisted approaches to omics data further enhances the identification of molecular targets for phenolic compounds and other phytochemicals, facilitating the rational design of nutraceutical interventions tailored to specific health conditions [100]. By bridging the gap between preclinical and clinical research, omics technologies provide the mechanistic depth necessary to move beyond empirical observations towards a predictive and personalized approach to nutraceutical development. The integration of artificial intelligence with nutrigenomics and multi-omics data is advancing the field of personalized nutrition, enabling the identification of novel biomarkers, the prediction of individual responses to dietary interventions, and the development of tailored dietary recommendations [101]. AI-driven approaches are also being applied to integrate omics data with digital health technologies, creating a comprehensive framework for personalized nutrition that considers genetic, metabolic, and lifestyle factors [102].

### 6.3. Personalized Nutrition

The concept of personalized nutrition represents a paradigm shift from the one-size-fits-all approach to dietary recommendations and nutraceutical interventions. This approach recognizes that individual responses to bioactive compounds are influenced by a complex interplay of genetic polymorphisms, epigenetic modifications, metabolic phenotypes, gut microbiome composition, and lifestyle factors, all of which contribute to substantial inter-individual variability in efficacy and safety.

It is essential to define what distinguishes precision nutraceuticals from conventional standardized supplements or formulations with improved delivery systems. Conventional nutraceuticals aim to deliver a fixed dose of a bioactive compound to a general population, with standardization focused on chemical consistency and enhanced bioavailability achieved through formulation technologies [103]. Precision nutraceuticals, by contrast, are constituted through the integration of individual-specific biological data, including genetic polymorphisms (e.g., BCMO1 variants influencing carotenoid absorption; single nucleotide polymorphisms affecting nutrient metabolism) [104], metabolic phenotypes (e.g., insulin sensitivity, lipid profiles), and gut microbiome composition (e.g., capacity to convert ellagitannins to urolithins) [105,106], to select the most appropriate bioactive compound, dose, and formulation for each individual. This approach requires a multi-step process: (i) assessment of individual biological characteristics; (ii) interpretation of these data to identify responder profiles; (iii) selection of personalized intervention; and (iv) monitoring of response and adjustment [2]. The key distinction is that precision nutraceuticals are not merely ‘personalized’ in terms of delivery or dosage, but are fundamentally informed by measurable determinants of individual response. However, it must be emphasized that this concept remains largely prospective, and clinical validation of precision nutraceutical interventions is still in its infancy.

The integration of individual omics profiles with clinical and dietary data enables the stratification of populations based on their predicted responsiveness to specific nutraceutical interventions, allowing for the selection of the most appropriate compounds, doses, and formulations for each individual [99]. Advanced delivery systems, such as nanoliposomes and plant-derived exosomes, are instrumental in realizing the potential of personalized nutrition, as they can be engineered to encapsulate and protect bioactive compounds, improve their bioavailability, and enable targeted release at specific sites of action [107]. Plant-derived exosomes have attracted considerable interest as natural nanocarriers, although their clinical application in precision nutrition remains largely speculative at this stage. While preclinical studies suggest potential for encapsulation and targeted delivery, robust evidence from human studies is currently lacking [108]. The combination of personalized profiling with advanced delivery systems may contribute to optimizing the potential benefits of agro-food-derived bioactives while minimizing the risks of adverse effects and ineffective interventions. The application of nanocarrier systems, including nanoliposomes and plant-derived exosomes, represents a promising strategy to overcome the bioavailability limitations of bioactive compounds and to enable their targeted delivery to specific tissues, thereby enhancing their therapeutic efficacy and reducing off-target effects [109].

### 6.4. Regulatory Challenges

The translation of agro-food by-products into commercial nutraceutical products is heavily dependent on the regulatory framework governing their production, marketing, and health claims. In the European Union, the regulatory landscape is particularly complex, with multiple layers of legislation that must be navigated to bring a product to market. The Novel Food Regulation (EU) 2015/2283 requires a comprehensive safety assessment for any ingredient that was not consumed to a significant degree in the EU before May 1997, including many compounds derived from agro-food by-products that are not considered traditional foods. The updated EFSA guidance on the safety assessment of novel foods emphasizes the need for robust toxicological data, including allergenicity risk assessment, which is particularly challenging for complex plant extracts that may contain multiple allergenic proteins or cross-reactive epitopes [110]. Furthermore, the Nutrition and Health Claims Regulation (EC) 1924/2006 imposes strict requirements for the scientific substantiation of any health-related claim, demanding that claims be based on generally accepted scientific evidence and be understood by the average consumer. The recent judgment of the European Court of Justice in Case C-386/23 (“Novel Nutriology”) addresses a distinct regulatory issue. Its central question concerns whether health claims relating to botanical substances, for which evaluation has been suspended by the European Commission, may be used in commercial advertising under the transitional provisions of Article 28(5) and (6) of Regulation (EC) No 1924/2006. The Court ruled that such claims are not permitted until the Commission has completed its examination for inclusion in the authorized lists, unless specifically permitted under Article 28(6). This judgment therefore concerns the applicability of transitional measures for pending botanical claims, rather than establishing criteria for causal substantiation through human intervention studies. The distinction between ingredient safety authorization, health-claim authorization, and evidence of therapeutic efficacy must be maintained [111].

Several categories of ingredients derived from agro-food by-products can be distinguished. ‘Traditional foods’ from third countries may be notified under simplified procedures if a history of safe use is documented [112]. ‘Novel foods’ require a comprehensive safety assessment under Regulation (EU) 2015/2283 [113]. ‘Food supplements’ are regulated as concentrated sources of nutrients or other substances with a nutritional or physiological effect [114]. ‘Nutraceuticals’ is a marketing term without a formal regulatory definition in EU law [114,115]. Ingredients most likely to face regulatory hurdles include those derived from by-products not traditionally consumed in the EU (e.g., pomegranate peel extracts, olive pomace concentrates) and those for which health claims are sought, as these require EFSA authorization based on generally accepted scientific evidence [111]. Table 4 provides a comparative overview of the regulatory frameworks for nutraceuticals and food supplements across major jurisdictions, highlighting the substantial heterogeneity in pre-market approval requirements, health claim substantiation, and classification criteria.

As illustrated in Table 4, the regulatory framework for nutraceuticals is characterized by significant heterogeneity across jurisdictions. The European Union and Canada require pre-market approval for most products [111,120], whereas the United States relies primarily on post-market surveillance [116]. Japan operates a dual system combining notification and approval pathways [118], while Australia and New Zealand employ a risk-based classification. This lack of international harmonization poses significant challenges for the commercialization of nutraceuticals derived from agro-food by-products, particularly for small and medium-sized enterprises seeking to access multiple markets [124].

The complexity and cost of meeting these regulatory requirements pose significant barriers to the commercialization of nutraceuticals derived from agro-food by-products, particularly for small and medium-sized enterprises with limited resources. Future progress will depend on the harmonization of regulatory frameworks across jurisdictions, the development of streamlined pathways for the approval of ingredients with a long history of safe use, and the establishment of public–private partnerships to support the generation of the scientific evidence required for claim substantiation. The regulatory landscape for nutraceuticals is characterized by significant heterogeneity across different jurisdictions, with the EU, USA, and Japan adopting distinct approaches to the classification, safety assessment, and health claim substantiation of these products, highlighting the need for greater international convergence to facilitate trade and consumer protection [125]. The EFSA guidance on botanicals and botanical preparations provides a comprehensive framework for safety assessment, addressing issues such as the characterization of botanical ingredients, the evaluation of toxicological data, and the assessment of potential interactions with conventional medicines [126]. The EFSA guidance on novel foods provides detailed requirements for the safety assessment of ingredients derived from non-traditional sources, including specific provisions for the evaluation of genotoxicity, reproductive toxicity, and chronic toxicity, which are particularly relevant for complex botanical extracts [113].

### 6.5. Integrated Strategies for the Future

Ultimately, the successful translation of agro-food-derived bioactives into clinically meaningful nutraceutical interventions will require an integrated approach that addresses the translational bottleneck at multiple levels simultaneously. This integration must encompass the development of standardized and traceable supply chains, the application of precision extraction technologies to produce consistent and bioactive-rich extracts, the use of advanced delivery systems to overcome bioavailability barriers, and the implementation of omics-based strategies to characterize individual responses and optimize interventions. Equally important is the engagement of regulatory authorities in the development of fit-for-purpose guidelines that recognize the unique characteristics of nutraceutical products, balancing the imperative of consumer safety with the need to foster innovation in the food and health sectors. The emerging field of precision nutrition, with its focus on tailoring interventions to individual metabolic profiles, offers a roadmap for the development of next-generation nutraceuticals that are not only effective but also safe and acceptable to consumers. By embracing the principles of sustainability, transparency, and evidence-based practice, the valorization of agro-food by-products can contribute to future health-promoting strategies, contributing to both human well-being and environmental sustainability. Based on the gaps identified in this review, we propose the following research priorities: (i) development of standardized and validated methods for the chemical characterization of by-product extracts, with a focus on identifying quality markers that correlate with biological activity; (ii) conduct of adequately powered, long-term clinical trials with clinically meaningful endpoints (e.g., cardiovascular events, cancer incidence) rather than intermediate biomarkers; (iii) elucidation of the mechanisms by which gut microbiota modulates the bioavailability and bioactivity of by-product-derived compounds, with a focus on inter-individual variability; (iv) development of precision delivery systems that can overcome bioavailability barriers and enable targeted release; (v) establishment of international regulatory harmonization for nutraceutical products derived from agro-food by-products; and (vi) integration of sustainability assessments (life-cycle analysis) into the development of by-product-derived nutraceuticals.

## Figures and Tables

**Table 1:** Main agro-food by-products, representative bioactive compounds, and associated biological activities.

Agro-Food Source	By-Product	Main Bioactive Compounds	Biological Activities	Evidence Level	References
Citrus	Peel, pulp, seeds	Flavanones (hesperidin, naringin, eriocitrin), polymethoxyflavones, hydroxycinnamic acids	Antioxidant, anti-inflammatory, cardioprotective, metabolic regulation	Preclinical (in vitro, animal); limited human data	[19,20,21,22,23]
Olive	Pomace	Hydroxytyrosol, tyrosol, oleuropein derivatives	Antioxidant, anti-inflammatory, cardioprotective, neuroprotective	Preclinical; some human evidence	[13,24]
Grape	Pomace	Anthocyanins, flavan-3-ols, proanthocyanidins, resveratrol	Antioxidant, anti-inflammatory, cardioprotective, neuroprotective	Preclinical; some human evidence	[25,26,27,28]
Tomato	Pomace	Lycopene, β-carotene, lutein, chlorogenic acid, flavonoids	Antioxidant, chemopreventive, anti-inflammatory, cardioprotective	Preclinical; human evidence for lycopene	[29,30,31,32]
Pomegranate	Peel	Ellagitannins, punicalagins, ellagic acid	Antioxidant, anti-inflammatory, antimicrobial, chemopreventive	Preclinical; limited human evidence	[33,34,35,36]

**Table 2:** Principal molecular mechanisms targeted by agro-food-derived bioactive compounds and representative phytochemicals.

Molecular Mechanism	Key Pathway	Biological Effect	Representative Compounds	Experimental Models	Evidence Level	References
Antioxidant Response	Nrf2/ARE	Upregulation of cytoprotective enzymes (HO-1, NQO1, GST), protection against oxidative stress	Resveratrol, curcumin, quercetin, anthocyanins, polymethoxyflavones	In vitro, animal	Preclinical	[50,51,52]
Inflammatory Signaling	NF-κB	Inhibition of pro-inflammatory cytokines (TNF-α, IL-1β, IL-6), suppression of COX-2 and iNOS	Polyphenols, flavonoids, hydroxytyrosol, punicalagin	In vitro, animal	Preclinical	[53,54,55]
Metabolic Regulation	AMPK/SIRT1/PGC-1α	Improvement of insulin sensitivity, fatty acid oxidation, mitochondrial biogenesis	Resveratrol, curcumin, flavonoids, hydroxytyrosol	In vitro, animal	Preclinical	[51,53,56]
Autophagy	FoxO3a/autophagy	Modulation of autophagic flux	Punicalagin	In vitro (macrophages)	Preclinical	[57]
Mitophagy	PINK1/Parkin	Clearance of damaged mitochondria, maintenance of mitochondrial quality control	Resveratrol, hydroxytyrosol, polyphenols, punicalagin	In vitro (macrophages)	Preclinical	[52,57,58,59]
Mitochondrial Homeostasis	Fusion/Fission, Biogenesis	Preservation of mitochondrial dynamics, ATP production, reduction in oxidative damage	Hydroxytyrosol, resveratrol, flavonoids, phenolic compounds	In vitro, animal	Preclinical	[51,60,61]
Apoptosis	Intrinsic/Extrinsic Pathways	Induction of apoptosis in cancer cells (caspase activation); protection against stress-induced cell death	Flavonoids, polyphenols, terpenoids, curcumin	In vitro	Preclinical	[53,62]

**Table 3:** Human intervention studies with preparations derived from agro-food by-products: study characteristics, outcomes, and limitations.

By-Product Source	Preparation and Provenance	Study Design	Population (*n*)	Dose and Duration	Comparator	Main Outcomes	Adverse Events	Limitations	References
Olive pomace	Olive pomace-enriched biscuit (17.1 mg/100 g hydroxytyrosol derivatives)	Randomized, double-blind, controlled parallel dietary intervention	Mildly hypercholesterolemic subjects (*n* = 62)	90 g biscuit/day, 8 weeks	Isoenergetic control biscuit	Trend towards reduced ox-LDL; ↑ phenolic acids in urine; ↑ homovanillic acid and DOPAC in blood	Not reported	Trend only for ox-LDL; sex effect observed	[67]
Olive pomace	Olive pâté tablets (30 mg/day hydroxytyrosol)	Randomized, crossover trial	Healthy adults (*n* = 19)	4 tablets/day (30 mg HT), 2 months	Placebo	↓ total cholesterol (−10.8 mg/dL), ↓ LDL (−10.8 mg/dL); ↑ Nrf-2 (+88.9%); ↓ MCP-1 (−9.0 pg/mL)	Not reported	Small sample; short duration	[68]
Grape pomace	Maltodextrinated grape pomace extract (MaGPE)	Randomized, placebo-controlled clinical trial	Diabetic retinopathy patients (*n* = 99)	6 months	Placebo	↑ BCVA; ↓ CRT; ↓ dROMs; ↓ oxLDL	Not reported	Specific population (DR patients)	[69]
Grape pomace	Dried grape pomace (8 g/day)	Randomized, crossover controlled trial	Subjects at cardiometabolic risk (*n* = 50)	8 g/day, 6 weeks	Control	↓ fasting insulinaemia (*p* < 0.01); trend for postprandial insulin	Not reported	Only insulin sensitivity affected	[70]
Tomato by-products	Processed tomato products (soup, juice)	Randomized clinical trial	Healthy adults (*n* = 60)	23–35 mg lycopene/day, 15 days	Lycopene-free diet washout	↑ plasma lycopene (57–123%); ↑ lipoprotein oxidation lag time	Not reported	Short duration; no clinical endpoints	[71]
Pomegranate peel	Pomegranate peel extract (500 mg twice daily)	Randomized, double-blind, placebo-controlled	Women with knee osteoarthritis (*n* = 60)	500 mg twice daily, 8 weeks	Placebo	↓ hs-CRP, ↓ NF-κB, ↓ MMP1, ↓ MCP-1	Not reported	Specific population (knee OA)	[72]
Pomegranate peel	Pomegranate peel extract	Randomized, double-blind clinical trial	NAFLD patients	Not specified	Placebo	Amelioration of metabolic syndrome risk factors	Not reported	NAFLD-specific	[73]
Citrus by-products	Citrus extract high in flavonoids (500 mg/day)	Randomized, placebo-controlled	Subjects with metabolic syndrome features	500 mg/day, 12 weeks	Placebo	Shift in SCFA profile towards butyrate; trend ↓ fecal calprotectin	Not reported	Focus on intestinal outcomes	[74]
Citrus by-products	Standardized citrus flavonoid nutraceutical (Eriomin^®^; 70% eriocitrin, 5% hesperidin, 4% naringin)	Randomized, double-blind, placebo-controlled, crossover trial	Prediabetic patients on metformin (*n* = 71)	250 mg/day, 12 weeks per phase (2-week washout)	Placebo	↓ 2-h OGTT glucose (5%); preserved GLP-1; ↓ TNF-α (12%); ↑ FRAP (7.5%); ↓ body weight, fat mass, BMI; ↓ systolic BP (4%)	Well tolerated; 9 GI events, mostly in metformin-naïve participants; 3 withdrawals	Short duration; specific population (prediabetic on metformin)	[65]
Citrus bergamot	Bergamot polyphenolic fraction (BPF)	Randomized, double-blind, placebo-controlled	Type 2 diabetes with hyperlipemia (*n* = 60)	BPF or BPF Phyto, duration not specified	Placebo	↓ fasting glucose, ↓ LDL, ↓ triglycerides; ↑ HDL	Not reported	Small sample	[75]

Note: ↑, increase; ↓, decrease.

**Table 4:** Comparative regulatory frameworks for nutraceuticals and food supplements.

Jurisdiction	Regulatory Framework	Pre-market Approval	Key Features	References
European Union	Regulation (EC) 1924/2006; Regulation (EU) 2015/2283	Health claims require EFSA authorization; novel foods require safety assessment	Distinction between food supplements and medicinal products; no legal definition of “nutraceuticals”	[111,112,113,115]
United States	Dietary Supplement Health and Education Act (DSHEA, 1994)	No pre-market approval required for most products; New Dietary Ingredient (NDI) notification 75 days before marketing	Manufacturer responsible for safety; structure/function claims allowed with disclaimer	[116,117]
Japan	Health Promotion Law; Food with Functional Claims (FFC) system	FFC: 60-day pre-market notification; FOSHU: pre-market approval	Dual system: FFC (notification) and FOSHU (approval); strict safety and efficacy review for FOSHU	[118,119]
Canada	Natural Health Products Regulations (2003)	Pre-market approval required; Natural Product Number (NPN) assigned	Product and site licenses required; monographs for simplified applications	[120,121]
Australia/New Zealand	Therapeutic Goods Act 1989; Dietary Supplements Regulations	Listed medicines: pre-market assessment; Registered medicines: full evaluation	Risk-based classification (Listed vs. Registered); GMP compliance required	[122,123]
International Comparison	Multiple frameworks	Variable	Heterogeneity across jurisdictions; need for harmonization	[124]

## Data Availability

No new data were created or analyzed in this study. Data sharing is not applicable to this article.

## Abbreviations

The following abbreviations are used in this manuscript:

AI	Artificial Intelligence
AMPK	AMP-Activated Protein Kinase
ARE	Antioxidant Response Element
COX-2	Cyclooxygenase-2
DES	Deep Eutectic Solvents
EFSA	European Food Safety Authority
FAO	Food and Agriculture Organization
FoxO	Forkhead Box O
GST	Glutathione S-Transferase
HO-1	Heme Oxygenase-1
HPLC	High-Performance Liquid Chromatography
HPTLC	High-Performance Thin-Layer Chromatography
IκB	Inhibitor of κB
IKK	IκB Kinase
IL-1β	Interleukin-1β
iNOS	Inducible Nitric Oxide Synthase
Keap1	Kelch-like ECH-Associated Protein 1
LPS	Lipopolysaccharide
NADES	Natural Deep Eutectic Solvents
NF-κB	Nuclear Factor Kappa-Light-Chain-Enhancer of Activated B Cells
NQO1	NAD(P)H:Quinone Oxidoreductase 1
Nrf2	Nuclear Factor Erythroid 2-Related Factor 2
PGC-1α	Peroxisome Proliferator-Activated Receptor Gamma Coactivator 1-Alpha
PI3K	Phosphoinositide 3-Kinase
PINK1	PTEN-Induced Kinase 1
SIRT1	Sirtuin 1
TNF-α	Tumor Necrosis Factor-α
